# Going Abroad

**Published:** 1871-04

**Authors:** 


### GOING ABROAD.

MANY contemplate going abroad for their health, for pleasure, recreation, and relaxation. The cost of travel may interest those who design crossing the water. Steamers leave New York City for European ports every week-day except Monday. The Anchor line of steamers leave for Glasgow every Saturday; cabin passage from sixty to seventy-five dollars, according to the size and situation of the berth. Glasgow is a centre from which excursions can be made in a few hours in different directions to the

"BANKS AND BRAES OF BONNY DOON,"

to the Giant's Causeway in Ireland, to Belfast, the great place of manufacture of Irish linen, to some of the most beautiful of the lakes of Scotland, to Edinburgh, and to various castles of renown. A dozen dollars will take one by sea, or through the whole length of England, down to London, from Glasgow or Edinburgh; from the latter place ten dollars takes one to Rotterdam, by steamer; another ten pays for steaming up the length of the navigable Rhine to Manheim, within half an hour of picturesque old Heidelberg, where the traveller can remain several weeks with new daily delights and wonderments, at less than a gold dollar a day, at Herr Lang's. Ladies will find a very pleasant home at 58 Anlage, the promenade of the place, where Frau Fürster will make them cozy and comfortable as long as they choose to sit at her family table. From Heidelberg to the various watering places of Germany the traveller can go by rail in a few days, and gradually make his way southward to Switzerland and Italy.

Steamers leave New York for Hamburg direct every Tuesday, at one hundred and thirty dollars; four times a week for Liverpool, at prices varying from seventy to one hundred and thirty dollars.

